# Updating specificity for the urgent design of an Andes hantavirus RT-qPCR assay

**DOI:** 10.1016/j.nmni.2026.101799

**Published:** 2026-06-18

**Authors:** Antonio Martínez-Murcia, Aaron Navarro, Caridad Miró-Pina, Adrián García-Sirera, Laura Pérez

**Affiliations:** a Department of Microbiology, University Miguel Hernández, Orihuela, Alicante, 03312, Spain; b Genetic PCR Solutions®, Orihuela, Alicante, 03300, Spain

**Keywords:** Andes hantavirus, RT-qPCR, Specificity

Dear Editor,

On 6 May 2026, the World Health Organization confirmed Andes hantavirus (ANDV) as the causative agent of the cruise ship respiratory outbreak, highlighting the need for specific molecular diagnostics. Surprisingly, we were unable to identify available RT-qPCR kits specifically intended for ANDV detection. Commercial RT-qPCR assays were mainly designed for broad detection of the genus *Orthohantavirus* or for hantavirus pulmonary syndrome-causing hantaviruses. They frequently rely on highly degenerated primers to maximize species coverage, often compromising the inclusivity and sensitivity for specific targets. For example, the pan-orthohantavirus assay described by Goodfellow et al. (2022) [1] contains 11 degenerate positions between forward and reverse primers, substantially reducing the effective matching in primers hybridization and potentially affecting amplification efficiency across diverse ANDV strains.

Several publications describe RT-qPCR assays targeting ANDV [[2], [3], [4]]. We evaluated the *in silico* specificity of these assays against all publicly available *Orthohantavirus andesense* sequences deposited in NCBI GenBank. The assay of Kramski et al. (2007) [2], designed for simultaneous detection of ANDV and Sin Nombre virus, showed limited inclusivity: 15.60% of sequences showed five mismatches in the reverse primer, while 11.35% presented three to four mismatches in the same oligonucleotide. The assay of Nunes et al. (2019) [3], designed for Amazonian hantaviruses, showed lower inclusivity. A total of 72.86% sequences presented four to six mismatches in the probe region, 23.57% presented three probe mismatches, and 32.08% showed three mismatches in the reverse primer. The assay of Safronetz et al. (2009) [4], designed for specific ANDV detection, showed the highest inclusivity. Only 4.0% of sequences contained three mismatches in the forward primer and 3.73% contained three to four mismatches in the probe. However, exclusivity analysis predicted potential cross-reactivity with *Orthohantavirus maporalense* and *Orthohantavirus mamorense*, which exhibited only one to two mismatches in the forward primer region.

Given the scarcity of ANDV-specific assays, on 6 May 2026 we designed a new RT-qPCR assay to improve both inclusivity and exclusivity. *In silico* analysis of newly designed primers and probe, containing only a single degenerate nucleotide, showed inclusivity for approximately 97% of available sequences. The only three sequences not meeting this criterion, all containing three mismatches in the forward primer, corresponded to ANDV strain VARS/22-01 (18-JAN-2023), Pergamino virus strain 14403 (26-MAR-2002) and Maciel virus strain 13796 (26-MAR-2002), which are relatively distant from the major *Orthohantavirus andesense* phylogenetic clusters (Fig. 1a). Moreover, the assay showed full match with the first published sequence associated with the 2026 outbreak (ANDV/Switzerland/Hu-3337/2026). In contrast, previously published assays showed mismatches with this sequence, particularly Nunes et al. (2019) assay exhibited five mismatches across primers and probe. Additionally, evaluation of the newly designed assay against the 938 *Orthohantavirus andesense* sequences available in the BV-BRC database confirmed that they can all be detected. The assay was additionally calibrated *in vitro* using a synthetic DNA fragment containing the target region. A standard curve generated from ten-fold dilutions (10^6^ to 10 copies) showed linear amplification across the dynamic range (Fig. 1b). Reproducibility and repeatability (n = 10) were tested following UNE-EN ISO/IEC 17025:2005 criteria as by Bru et al. (2023) [5]. The protocol requires 28 min of cycling plus instrument ramping time, corresponding to approximately 45 min on current qPCR platforms. The assay became available on 13 May 2026 through a biotech-based company and has been subjected to evaluation by a reference laboratory using clinical samples related to the 2026 outbreak.

**Fig. 1:**
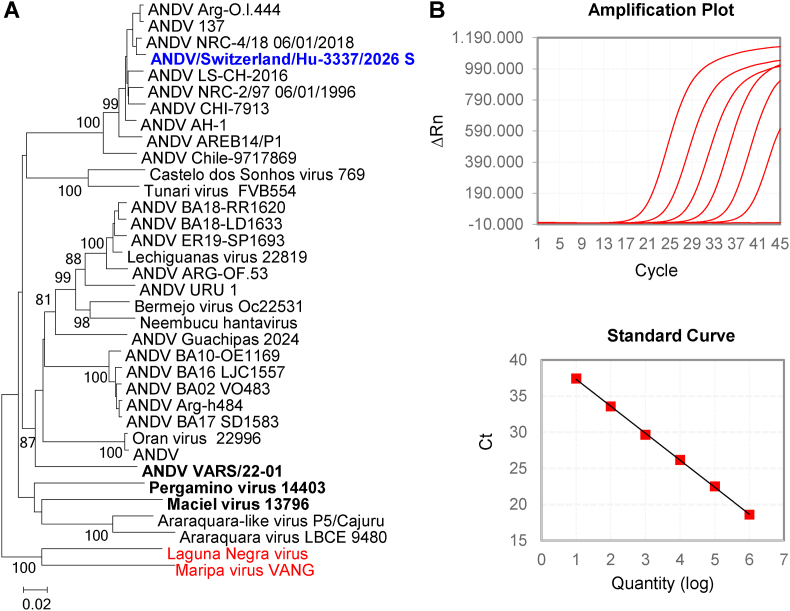
(A) Phylogenetic analysis of *Orthohantavirus andesense* based on the S-segment nucleocapsid (N) gene (neighbor-joining method; 1067 bp). Laguna Negra virus and Maripa virus (*Orthohantavirus mamorense*), in red, were used as outgroups. The first published sequence associated with the 2026 outbreak (ANDV/Switzerland/Hu-3337/2026) is highlighted in blue, and sequences discussed in the text are shown in bold. Numbers at nodes indicate bootstrap values (percentage of 1000 replicates). (B) Amplification plot and standard curve of the ANDV RT-qPCR assay generated using a synthetic DNA target from 10^6^ to 10 copies.

## Ethical approval

Not applicable.

## Funding

No funding has been received for this work.

## CRediT authorship contribution statement

**Antonio Martínez-Murcia:** Conceptualization, Formal analysis, Writing – original draft, Writing – review & editing. **Aaron Navarro:** Conceptualization, Formal analysis, Writing – original draft, Writing – review & editing. **Caridad Miró-Pina:** Conceptualization, Formal analysis, Writing – original draft, Writing – review & editing. **Adrián García-Sirera:** Conceptualization, Formal analysis, Writing – original draft, Writing – review & editing. **Laura Pérez:** Conceptualization, Formal analysis, Writing – original draft, Writing – review & editing.

## Declaration of competing interest

Antonio Martínez-Murcia reports a relationship with genetic PCR solutions that includes: consulting or advisory. Aaron Navarro reports a relationship with genetic PCR solutions that includes: employment. Caridad Miró-Pina reports a relationship with genetic PCR solutions that includes: employment. Adrián García-Sirera reports a relationship with genetic PCR solutions that includes: employment. Laura Pérez reports a relationship with genetic PCR solutions that includes: employment. If there are other authors, they declare that they have no known competing financial interests or personal relationships that could have appeared to influence the work reported in this paper.
